# Supplementary material for: Transcriptome Analysis Reveals Vimentin-Induced Disruption of Cell–Cell Associations Augments Breast Cancer Cell Migration
Source: Cells. 2022 Dec 13;11(24):4035. doi: 10.3390/cells11244035 (PMC9776984; doi:10.3390/cells11244035)
Supplement: Supplementary file 1 [file cells-11-04035-s001.zip › DEGs and enrichment analysis.zip!/DEGs and enrichment analysis/WT_VIMvsVector KEGG/DOWN/WT_VIMvsVector_DOWN.pathway_map.zip!/WT_VIMvsVector_DOWN.pathway_map/src/hsa04010.html]

hsa04010


- hsa:1846

- Down regulated genes

ENSG00000120875(-1.53539746240952)

- hsa:25780

- Down regulated genes

ENSG00000152689(-4.14165052550698)

- hsa:3791

- Down regulated genes

ENSG00000128052(-2.65973347544558)

- hsa:1846

- Down regulated genes

ENSG00000120875(-1.53539746240952)

- hsa:7042

- Down regulated genes

ENSG00000092969(-2.32734226266555)

- hsa:374

- Down regulated genes

ENSG00000109321(-2.16481325315589)

Close
